# NLRC5 shields T lymphocytes from NK-cell-mediated elimination under inflammatory conditions

**DOI:** 10.1038/ncomms10554

**Published:** 2016-02-10

**Authors:** Kristina Ludigs, Camilla Jandus, Daniel T. Utzschneider, Francesco Staehli, Stéphanie Bessoles, Anh Thu Dang, Giorgia Rota, Wilson Castro, Dietmar Zehn, Eric Vivier, Werner Held, Pedro Romero, Greta Guarda

**Affiliations:** 1 Department of Biochemistry, University of Lausanne, Epalinges 1066, Switzerland; 2 Ludwig Center for Cancer Research of the University of Lausanne, Epalinges 1066, Switzerland; 3 Division of Allergology and Immunology, CHUV, University of Lausanne, Epalinges 1066, Switzerland; 4 Centre d'Immunologie de Marseille-Luminy, Aix-Marseille University UM2, Inserm U1104, CNRS UMR7280, Marseille 13288, France; 5 Immunologie, Hôpital de la Conception, Assistance Publique—Hôpitaux de Marseille, Marseille 13385, France

## Abstract

NLRC5 is a transcriptional regulator of MHC class I (MHCI), which maintains high MHCI expression particularly in T cells. Recent evidence highlights an important NK–T-cell crosstalk, raising the question on whether NLRC5 specifically modulates this interaction. Here we show that NK cells from *Nlrc5*-deficient mice exhibit moderate alterations in inhibitory receptor expression and responsiveness. Interestingly, NLRC5 expression in T cells is required to protect them from NK-cell-mediated elimination upon inflammation. Using T-cell-specific *Nlrc5*-deficient mice, we show that NK cells surprisingly break tolerance even towards ‘self' *Nlrc5*-deficient T cells under inflammatory conditions. Furthermore, during chronic LCMV infection, the total CD8^+^ T-cell population is severely decreased in these mice, a phenotype reverted by NK-cell depletion. These findings strongly suggest that endogenous T cells with low MHCI expression become NK-cell targets, having thus important implications for T-cell responses in naturally or therapeutically induced inflammatory conditions.

## Abstract (web summary)

NK cell tolerance to self-MHCI levels is calibrated during their development. Here the authors show that this tolerance is overcome by an inflammatory environment and that NLRC5 protects T cells from NK cell-mediated elimination by maintaining high MHCI expression.

## Graphical abstract

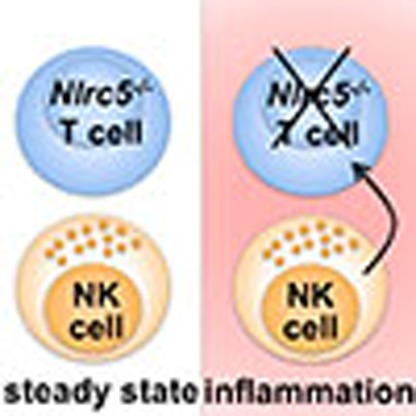

Major histocompatibility complex class I (MHCI) molecules are ubiquitously expressed surface glycoproteins, crucial for the function of CD8^+^ T and natural killer (NK) cells. These two cytotoxic lymphocyte subsets mediate immunity towards infected or transformed cells using complementary recognition strategies. Cells presenting foreign antigens in MHCI will be recognized and killed by CD8^+^ T cells. Conversely, NK lymphocytes eliminate cells lacking MHCI expression, a phenomenon known as ‘missing-self recognition'.

Besides NF-κB and interferon regulatory factors, NOD-like receptor (NLR) caspase recruitment domain containing protein 5 (NLRC5) has recently been identified as a key transcriptional regulator of MHCI genes^1^,^2^,^3^,^4^,^5^,^6^. As recently shown by chromatin immunoprecipitation sequencing, NLRC5 is exclusively dedicated to regulate the classical MHCI genes *H2-K* and *H2-D*, but also beta-2 microglobulin (*B2m*) and selected non-classical MHCI genes by occupying a specific SXY sequence in their promoter^2^. This NLR is constitutively expressed at high levels in immune cells and predominantly in lymphocytes^1^. Accordingly, its deficiency moderately affects MHCI expression in conventional dendritic cells and macrophages, whereas a strong decrease is observed in lymphocytes, with T cells displaying the most prominent defect^1^,^2^,^5^.

The relevance of MHCI expression on T cells remains poorly explored. Interestingly, several studies highlighted an important crosstalk between T and NK cells, particularly in viral infections^7^,^8^,^9^,^10^,^11^,^12^,^13^,^14^,^15^,^16^,^17^,^18^. A recent report suggested that high levels of MHCI on CD8^+^ T cells are essential to protect them from NK cell-dependent elimination during antiviral responses^15^. In more detail, antiviral CD8^+^ T cells deficient for type-I interferon receptor (*Ifnar*) were rejected by NK cells following infection. Interestingly, the authors detected a very low expression of classical and non-classical MHCI on *Ifnar*^*−/−*^ T cells as compared with their wild-type counterparts, suggesting a straightforward explanation for eliciting NK-cell rejection^15^.

NK cells acquire the ability to discriminate normal from absent MHCI levels through a process known as NK-cell education, which is dictated by the engagement of inhibitory receptors by MHCI ligands. Indeed, NK cells derived from MHCI-deficient *B2m* knockout mice or from mice lacking the phosphatase SHP-1, a key signalling molecule downstream of MHCI receptors, are hyporesponsive^19^,^20^,^21^,^22^. A direct correlation exists between the level of inhibitory receptor engagement by MHCI molecules and NK-cell responsiveness, as shown using MHCI heterozygous and transgenic mice^23^,^24^,^25^. Although it is unclear what minimal level of MHCI is needed to establish NK-cell reactivity and to ensure tolerance, the presence of a sizable population of MHCI-negative cells prevents ‘missing-self' reactivity^24^,^26^,^27^,^28^. Responsiveness of NK cells is therefore thought to be tuned to endogenous levels of MHCI and the presence of MHCI-negative cells dominantly establishes tolerance.

Why NLRC5 evolved to control MHCI transcription in lymphocytes and, most prominently, in T cells remained unclear. The emerging evidence interconnecting NK- and T-cell responses led us to hypothesize that NLRC5-dependent expression of MHCI might be critical for regulating this crosstalk. We therefore set off to evaluate the impact of *Nlrc5* deficiency in T cells on the interactions of these two cell subsets.

On the one hand, we show here that NLRC5 plays a key role in protecting T cells from NK-cell-mediated elimination under inflammatory conditions, as demonstrated by the rejection of *Nlrc5*^*−/−*^ T cells upon transfer into Poly(I:C)-pretreated or infected mice. On the other hand, NK cells from *Nlrc5*-deficient or CD4cre *Nlrc5*^*fl/fl*^ mice (with selective *Nlrc5* deficiency in T cells) are surprisingly efficient in rejecting MHCI-negative cells, indicating that these animals host-responsive NK cells together with potential T-cell targets. Indeed, NK-cell-dependent loss of *Nlrc5*-deficient T cells is observed in CD4cre *Nlrc5*^*fl/fl*^ mice following Poly(I:C) pretreatment or viral infection. This suggests that tolerance to low MHCI levels can be overcome by an inflammatory environment, and that NLRC5 plays a key role in protecting T cells from NK-cell-mediated elimination under such conditions.

## Results

### *Nlrc5* ^*−/−*^ T cells display low but not absent MHCI levels

**Figure 1:**
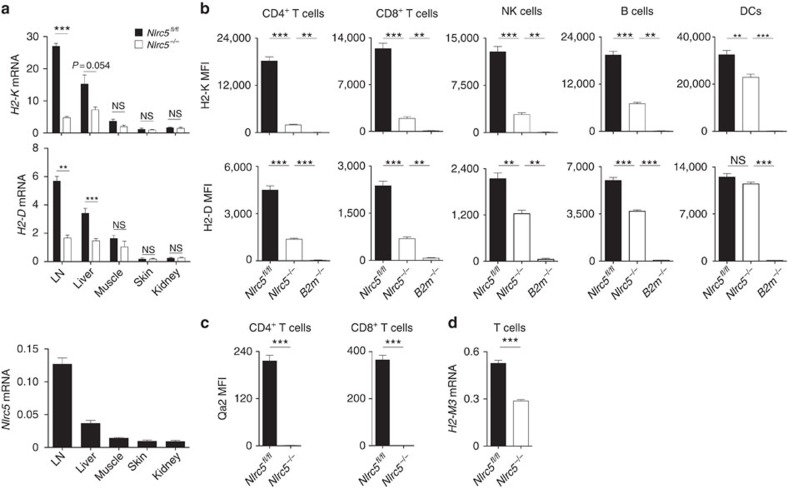
*Nlrc5*^*−/−*^ lymphocytes exhibit low MHCI expression. (**a**) qRT–PCR analysis (normalized to *Hprt*) in the indicated tissues is shown for *H2-K* and *H2-D* mRNA in *Nlrc5*^*fl/fl*^ and *Nlrc5*^*−/−*^ mice, and for *Nlrc5* mRNA in *Nlrc5*^*fl/fl*^ mice. Results represent mean±s.e.m. (*n*=3 mice per group). (**b**) H2-K and H2-D expression, depicted as mean fluorescence intensity (MFI), was analysed by flow cytometry on splenic CD4^+^ and CD8^+^ T cells (CD3^+^CD4^+^ and CD3^+^ CD8^+^, respectively), NK cells (NK1.1^+^CD3^−^), B cells (CD19^+^) and DCs (CD11c^high^) from *Nlrc5*^*fl/fl*^, *Nlrc5*^*−/−*^ and *B2m*^*−/−*^ mice. (**c**) Qa2 expression, depicted as MFI, was analysed on splenic CD4^+^ and CD8^+^ T cells. Results represent mean±s.e.m. (*n*=3–5) and are representative of at least three experiments (**b**,**c**). (**d**) *H2-M3* mRNA expression was quantified relative to *Hprt* mRNA in T cells purified from in *Nlrc5*^*fl/fl*^ and *Nlrc5*^*−/−*^ mice. Results depict mean±s.d. (*n*=3 replicates) and are representative of at least two experiments. NS, non-significant; **P<0.01; ***P<0.001; Student's *t*-test.

We and others have previously shown that NLRC5 regulates the expression of H2-K and H2-D in most immune cells and particularly in lymphocytes^1^,^4^,^5^. Our understanding of NLRC5 contribution to MHCI expression in non-immune tissues is however still incomplete^3^,^6^,^29^. We therefore analysed by quantitative real-time PCR (qPCR) *H2-K* and *H2-D* alongside with *Nlrc5* messenger RNA (mRNA) abundance in different tissues derived from control or *Nlrc5*-deficient mice. As shown in Fig. 1a, both MHCI and *Nlrc5* genes are expressed at lower levels in non-lymphoid tissues and, at steady state, NLRC5 does not contribute to MHCI transcription in organs such as skin and kidney. Among immune cells, the contribution by NLRC5 to MHCI expression varies in different cell subsets, with T cells exhibiting the major defect (Fig. 1b)^1^,^4^,^5^. In fact, these lymphocytes express on average 20% of the wild-type levels, having thus low residual expression of classical MHCI, H2-K and H2-D, as shown by comparison with *B2m*-deficiency (Fig. 1b). As we were interested in the role of NLRC5 in T cells, we further analysed the non-classical MHCI Qa2, recently shown by us to be a prime target of NLRC5 (ref. 2), and H2-M3. In agreement with previous reports, Qa2 was totally absent, whereas *H2-M3* mRNA was reduced to about half in *Nlrc5*-deficient T cells^2^,^4^ (Fig. 1c,d). Moreover, the classical MHCI molecule H2-L in *Nlrc5*^*−/−*^ BALB/c mice was reduced similarly to H2-K and -D on T lymphocytes (Supplementary Fig. 1a), indicating that also this MHCI gene is a target of NLRC5. Thus, *Nlrc5*-deficient mice present a complex mosaic in which MHCI is low but not absent on lymphocytes and normal on other tissues.

To gain insights into the extent of variation of NLRC5 and MHCI expression in humans, we tested the abundance of *NLRC5*, *HLA-B* and *HLA-C* mRNA in healthy donor-derived T cells. As shown in Supplementary Fig. 1b, expression of NLRC5 correlated with *HLA* gene expression, substantiating the role of NLRC5 in HLA transcriptional regulation^3^ and suggesting considerable interindividual variation in the expression of these genes, a phenomenon that can be mimicked by *Nlrc5* deficiency.

### *Nlrc5* deletion mildly alters Ly49I expression

**Figure 2:**
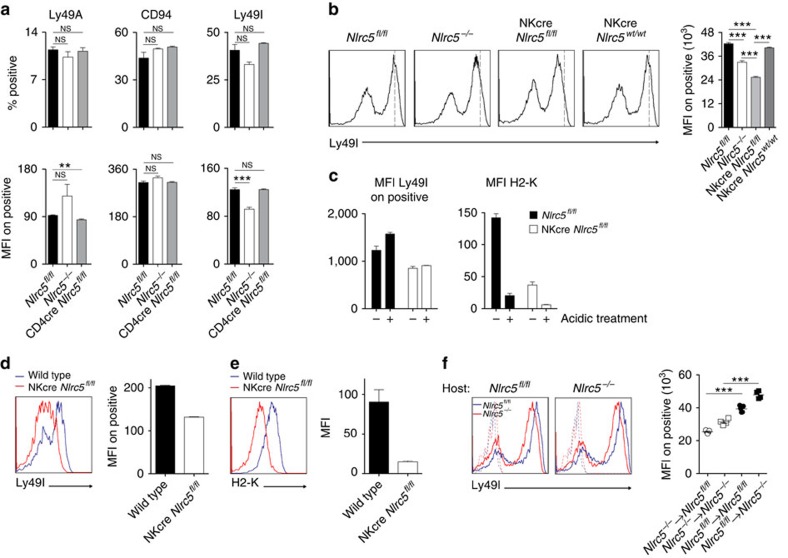
NK cells from *Nlrc5*^*−/−*^ and NKcre *Nlrc5*^*fl/fl*^ mice exhibit mildly decreased Ly49I expression. (**a**) Graphs depict percentages of Ly49A^+^, CD94^+^ and Ly49I^+^ NK cells, and MFI of Ly49A, CD94 and Ly49I of the positive population. (**b**) Histograms show Ly49I expression on NK cells from a representative sample of *Nlrc5*^*fl/fl*^, *Nlrc5*^*−/−*^, NKcre *Nlrc5*^*fl/fl*^ and NKcre *Nlrc5*^*wt/wt*^mice. Bar graphs depict the MFI of Ly49I on Ly49I^+^ NK cells. (**c**) Splenocytes from *Nlrc5*^*fl/fl*^ and NKcre *Nlrc5*^*fl/fl*^ mice were acid treated or not and analysed by flow cytometry. Graphs depict the MFI of Ly49I on Ly49I^+^ NK cells and H2-K on NK cells. Results represent mean±s.e.m. (*n*=3–4 mice per group) and are representative of three independent experiments (**a**–**c**). (**d**,**e**) Wild type:NKcre *Nlrc5*^*fl/fl*^ mixed BM chimeras were analysed at day 70 after reconstitution. Histograms show the expression of Ly49I (**d**) and H2-K (**e**) on NK cells as analysed on the indicated donor cells. Graphs show MFI of Ly49I on Ly49I^+^ NK cells (**d**) and MFI of H2-K on NK cells (**e**). (**f**) NK cells isolated from *Nlrc5*^*fl/fl*^ and *Nlrc5*^*−/−*^ mice were adoptively co-transferred into *Nlrc5*^*fl/fl*^ and *Nlrc5*^*−/−*^ hosts, and analysed for the expression of Ly49I 24 h after. Histograms illustrate the expression of Ly49I on the indicated donor cells (blue for *Nlrc5*^*fl/fl*^ and red for *Nlrc5*^*−/−*^ NK cells) and background fluorescence is shown in dashed lines (blue for *Nlrc5*^*fl/fl*^ and red for *Nlrc5*^*−/−*^ NK cells). Graphs show MFI of Ly49I on Ly49I^+^ NK cells. Results represent mean±s.e.m. (*n*=4–5 mice per group) and are representative of at least two independent experiments (**d**–**f**). NS, non-significant; **P<0.01; ***P<0.001; Student's *t*-test.

We next sought to phenotypically characterize NK cells from *Nlrc5*-deficient mice. Since we aimed at understanding how NLRC5 expression in T cells regulates the NK–T-cell crosstalk, we extended our analysis to NK cells from mice with specific *Nlrc5* ablation in T cells (CD4cre *Nlrc5*^*fl/fl*^, characterized in Supplementary Fig. 2a). Analyses of bone marrow (BM) and spleen did not reveal substantial alterations in NK-cell development and maturation in *Nlrc5*-deficient and CD4cre *Nlrc5*^*fl/fl*^ mice (Supplementary Fig. 2b,c). We next assessed the expression of NK-cell receptors specific for MHCI. Whereas NK cells derived from *B2m* knockout mice are known to exhibit higher levels of these receptors^30^, NK cells from *Nlrc5*^*−/−*^ and CD4cre *Nlrc5*^*fl/fl*^ mice expressed Ly49A and CD94 at normal levels (Fig. 2a). Unexpectedly, the intensity of Ly49I expression on Ly49I-positive cells was found to be decreased on NK cells from *Nlrc5*-deficient but not CD4cre *Nlrc5*^*fl/fl*^ mice (Fig. 2a). A similar trend was observed using an antibody recognizing Ly49C/I (Supplementary Fig. 2d,e)^31^. As the levels of Ly49C/I were affected by *Nlrc5* deficiency, we tested whether the educated Ly49C/I^+^ subset might express higher levels of NLRC5 (ref. 32). However, *Nlrc5* transcript abundance was equal in Ly49C/I^+^ and Ly49C/I^−^ subsets (Supplementary Fig. 2f; *H2-K* and *Ly49I* mRNA are here shown as controls). Likewise, human CD56^bright^ and CD56^dim^ NK cells expressed similar levels of *NLRC5* mRNA (Supplementary Fig. 2g), indicating that NLRC5 is broadly expressed among NK-cell subsets.

Compatibly with our observation that Ly49I was decreased in *Nlrc5*^*−/−*^ but not in CD4cre *Nlrc5*^*fl/fl*^ mice, *cis*-interactions with MHCI have recently been proposed to shape the repertoire of inhibitory receptors^26^. We therefore analysed Ly49I expression in mice specifically lacking *Nlrc5* in NK cells (NKcre *Nlrc5*^*fl/fl*^, characterized in Supplementary Fig. 2a)^33^. Remarkably, the defect in Ly49I expression was stronger than in *Nlrc5*^*−/−*^ mice (Fig. 2b) and not reversed by an acidic treatment (Fig. 2c; H2-K shown as control), indicating that MHCI-mediated masking was not responsible for the reduction^34^. Mixed wild type:NKcre *Nlrc5*^*fl/fl*^ BM chimeras showed that the reduction in Ly49I was NK-cell intrinsic (Fig. 2d; H2-K shown as control in Fig. 2e). To better dissect the underlying mechanisms, we adoptively transferred *Nlrc5*^*fl/fl*^ and *Nlrc5*^*−/−*^ NK cells into *Nlrc5*^*fl/fl*^ and *Nlrc5*^*−/−*^ recipient mice and analysed their expression of Ly49I 24 h after. As shown in Fig. 2f, we observed higher expression levels on NK cells transferred into *Nlrc5*^*−/−*^ than *Nlrc5*^*fl/fl*^ recipient mice and a greater defect on *Nlrc5*^*−/−*^ NK cells as compared with their *Nlrc5*^*fl/fl*^ counterparts. Taken together, these data show that MHCI levels in the environment inversely correlate to NK-cell inhibitory receptor expression and, unexpectedly, they also infer that *Nlrc5*-driven MHCI expression on NK cells exerts a positive influence on Ly49I levels.

### NK cells from *Nlrc5* ^*−/−*^ mice respond to MHCI-deficient cells

**Figure 3:**
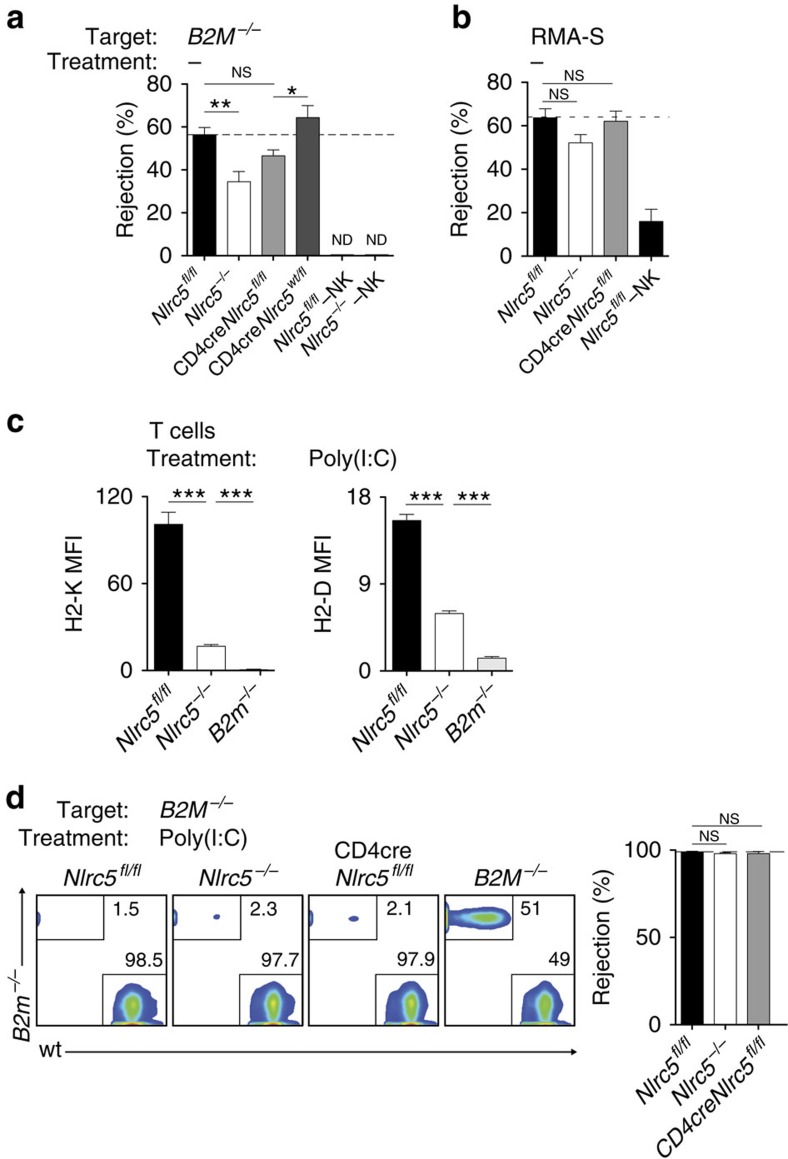
NK cells from *Nlrc5*^*−/−*^ and CD4cre *Nlrc5*^*fl/fl*^ mice efficiently respond to MHCI-deficient cells. (**a**) Rejection of *B2m*^*−/−*^ splenocytes was analysed by flow cytometry in the spleen of *Nlrc5*^*fl/fl*^, *Nlrc5*^*−/−*^, CD4cre *Nlrc5*^*fl/fl*^, CD4cre *Nlrc5*^*wt/fl*^, and NK cell-depleted *Nlrc5*^*fl/fl*^ and *Nlrc5*^*−/−*^ mice 2 days after transfer. As control, wild-type splenocytes were co-injected with *B2m*^*−/−*^ splenocytes. (**b**) *In vivo* tumour clearance was assessed at day 2 after intraperitoneal injection of RMA-S cells (normalized to co-injected RMA cells) in the peritoneal lavage of *Nlrc5*^*fl/fl*^, *Nlrc5*^*−/−*^, CD4cre *Nlrc5*^*fl/fl*^ and NK cell-depleted *Nlrc5*^*fl/fl*^ mice. (**c**) H2-K and H2-D expression, depicted as MFIs, were analysed on splenic T cells (CD3^+^) from *Nlrc5*^*fl/fl*^, *Nlrc5*^*−/−*^ and *B2m*^*−/−*^ mice 2 days after Poly(I:C) challenge. (**d**) Loss of *B2m*^*−/−*^ splenocytes was analysed in the spleen 1 day after transfer into *Nlrc5*^*fl/fl*^, *Nlrc5*^*−/−*^, CD4cre *Nlrc5*^*fl/fl*^ and *B2m*^*−/−*^ mice, which had been pretreated with Poly(I:C) 1 day before cell transfer. As control, wild-type splenocytes were co-injected. Results represent mean±s.e.m. of *n*=3–4 mice per group (**a**,**c**), *n*=5–7 mice per group (**b**) and *n*=3–6 mice per group (**d**), and are representative of at least two independent experiments (**a**–**d**). ND, not detected; −NK, depleted of NK cells; NS, non-significant; *P<0.05; **P<0.01; ***P<0.001; Student's *t*-test.

The functionality of NK cells from *Nlrc5*^*−/−*^ and CD4cre *Nlrc5*^*fl/fl*^ mice was then assessed by testing the ability to reject *B2m*^*−/−*^ splenocytes (Fig. 3a) or MHCI-negative RMA-S tumour cells (Fig. 3b). Surprisingly, both *Nlrc5*^*−/−*^ and CD4cre *Nlrc5*^*fl/fl*^ mice rejected the target cells, albeit *Nlrc5*^*−/−*^ mice in particular were less efficient as compared with control mice (Fig. 3a,b). To define the specific contribution by *Nlrc5* expression in NK cells to this outcome, we analysed the ability of NKcre *Nlrc5*^*fl/fl*^ to kill *B2m*^*−/−*^ splenocytes. However, no significant difference was observed (Supplementary Fig. 3), indicating that expression of NLRC5 in NK cells is *per se* not crucial to maintain NK-cell responsiveness.

Rejection of *B2m*-deficient splenocytes was then assessed in mice pretreated with Poly(I:C), which primes NK cells. Importantly, the reduced MHCI expression observed in *Nlrc5* knockout lymphocytes was maintained after Poly(I:C) pretreatment, exhibiting ∼30% residual expression of classical MHCI in *Nlrc5*^*−/−*^ T cells (Fig. 3c). A complete rejection was remarkably observed both in *Nlrc5*-deficient and CD4cre *Nlrc5*^*fl/fl*^ mice, but not in *B2m*-deficient mice (Fig. 3d). Thus, despite strongly reduced MHCI levels, NK cells from *Nlrc5*^*−/−*^ and CD4cre *Nlrc5*^*fl/fl*^ mice are surprisingly reactive to MHCI-deficient cells, indicating a nearly normal education state.

### NLRC5 protects T cells from NK-cell-mediated elimination

**Figure 4:**
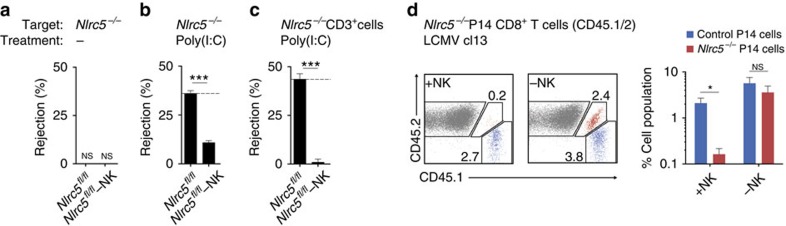
Transferred *Nlrc5*-deficient T cells are eliminated by NK cells under inflammatory conditions. (**a**) Elimination of *Nlrc5*^*−/−*^ splenocytes was analysed by flow cytometry in the spleen of *Nlrc5*^*fl/fl*^ and NK-cell-depleted *Nlrc5*^*fl/fl*^ mice 2 days after transfer. Wild-type splenocytes were co-injected as control. (**b**,**c**) *In vivo* elimination of *Nlrc5*^*−/−*^ total splenocytes (**b**) or gated on CD3^+^ T cells (**c**) was analysed in the spleen 1 day after transfer into *Nlrc5*^*fl/fl*^ and NK-cell-depleted *Nlrc5*^*fl/fl*^ mice, which had been pretreated with Poly(I:C) 1 day before cell transfer. Wild-type splenocytes were co-injected as control. Results represent mean±s.e.m. of *n*=4 mice per group (**a**) or *n*=4–6 mice per group (**b**,**c**) and are representative of at least two independent experiments (**a**–**c**). (**d**) *Nlrc5*^+/−^ (CD45.1^+^) and *Nlrc5*^*−/−*^ (CD45.1/2^+^) P14 CD8^+^ T cells were co-transferred into naive *Nlrc5*^*fl/fl*^ and NK-cell-depleted *Nlrc5*^*fl/fl*^ recipient mice, which were then infected with LCMV clone 13. Flow cytometric analysis of the spleen at day 8 post infection shows percentages of CD8^+^ cells stained with CD45.1 and CD45.2. The graph on the right represents mean±s.e.m. (*n*=5) mice per group and these results are representative of two independent experiments. ND, not detected; +NK, non-depleted; −NK, depleted of NK cells; NS, non-significant; *P<0.05; ***P<0.001; Student's *t*-test.

We proceeded to analyse whether NLRC5 deficiency in T lymphocytes influenced their rejection by NK cells. First, we assessed the susceptibility of *Nlrc5*-deficient splenocytes to NK-cell-mediated rejection upon transfer into wild-type recipients. Naive control mice failed to reject *Nlrc5*-deficient splenocytes (Fig. 4a), indicating that the low MHCI levels expressed by *Nlrc5*-deficient cells were sufficient to protect them from NK-cell-mediated attack. Instead, rejection of *Nlrc5*-deficient splenocytes (36%) was observed in mice pretreated with Poly(I:C) (Fig. 4b). We then took a closer look at *Nlrc5*-deficient T cells, which present the strongest defect in MHCI expression, and observed that their rejection was indeed superior (44%) and fully dependent on NK cells (Fig. 4c). These data demonstrate that NLRC5 is required to prevent NK-cell-mediated elimination of splenocytes, and T cells in particular, in Poly(I:C)-primed mice. To extend these findings into a more physiological situation, *Nlrc5*^*−/−*^ and control transgenic P14 T cells, which bear a T-cell receptor specific for the glycoprotein 33–41 (gp33) epitope of the lymphocytic choriomeningitis virus (LCMV), were co-transferred into recipient *Nlrc5*^*fl/fl*^ mice. These were then challenged with LCMV clone 13 and analysed 8 days post infection for the expansion of transferred cells (Fig. 4d). Whereas control P14 T cells nicely expanded, *Nlrc5*^*−/−*^ P14 T cells were nearly undetectable. Strikingly, the latter were rescued by NK-cell depletion (Fig. 4d). These results demonstrate that NLRC5 becomes essential to protect T cells against NK-cell-mediated rejection in inflammatory milieus and upon viral infection.

### NLRC5 maintains CD8^+^ T cells upon chronic LCMV infection

**Figure 5:**
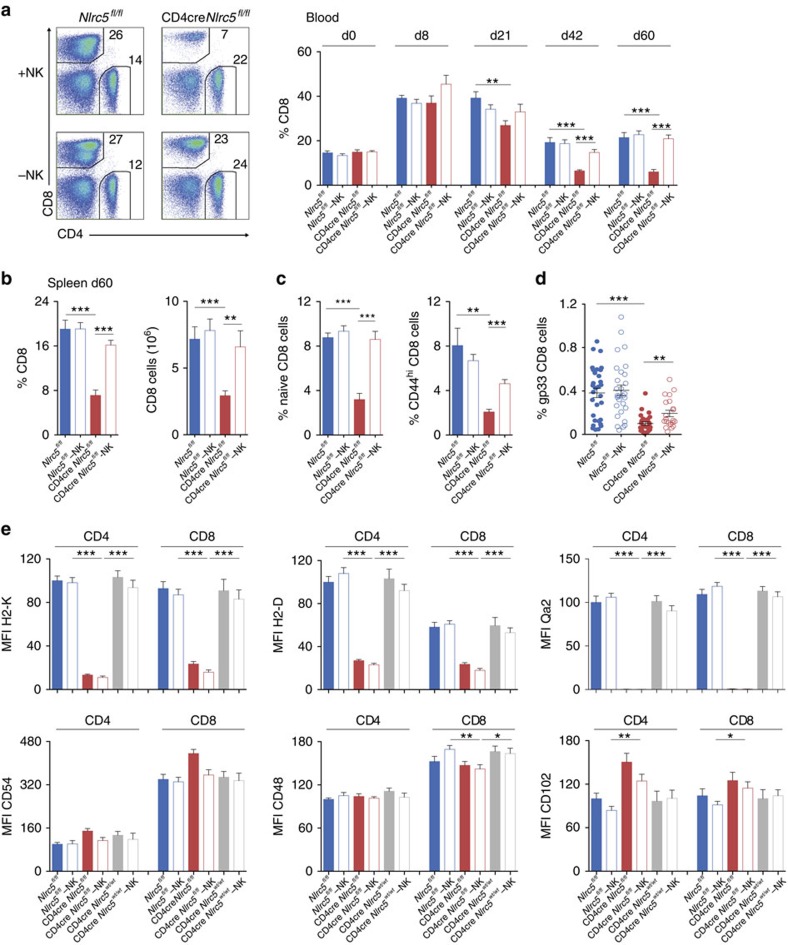
Loss of endogenous CD8^+^ T cells during LCMV infection in CD4cre *Nlrc5*^*fl/fl*^. (**a**) Percentages of CD8^+^ T cells (gated on lymphocytes) in the blood are illustrated by flow cytometric analysis at day 60 post infection and plotted over the course of LCMV clone 13 infection for NK cell-depleted or not *Nlrc5*^*fl/fl*^ and CD4cre *Nlrc5*^*fl/fl*^ mice. (**b**) Percentages and absolute numbers of splenic CD8^+^ T cells are depicted for day 60. (**c**) Percentages of naive (CD62L^hi^CD44^low^) and CD44^hi^ CD8^+^ T cells among total lymphocytes were analysed in the spleen at day 60 post infection. Results show a pool of two experiments representing mean±s.e.m. (*n*=6–13 mice per group) and are representative of at least three independent experiments (**a**–**d**) Percentages of virus-specific gp33^+^ CD8^+^ T cells were analysed in the spleen. Due to variation of this parameter, data show mean±s.e.m. (*n*=21–31 mice per group) and is a pool of five independent experiments. Statistical differences are depicted between CD4cre *Nlrc5*^*fl/fl*^ and *Nlrc5*^*fl/fl*^ or NK-depleted CD4cre *Nlrc5*^*fl/fl*^, respectively, when significant. (**e**) H2-K, H2-D, Qa2, CD54, CD48 and CD102 expression, shown as MFI (average MFI of control CD4 T cells mice was set at 100%), were analysed on splenic CD4^+^ and CD8^+^ T cells in the spleen of *Nlrc5*^*fl/fl*^, CD4cre *Nlrc5*^*fl/fl*^ and CD4cre *Nlrc5*^*wt/wt*^ mice, depleted of NK cells or not, infected for 48 days. Results represent mean±s.e.m. (*n*=6–10) and are a pool of two independent experiments. Only significant differences are depicted (**e**). +NK, non-depleted; −NK, depleted of NK cells; *P<0.05; **P<0.01; ***P<0.001; Student's *t*-test.

Since NK cells from CD4cre *Nlrc5*^*fl/fl*^ and *Nlrc5*-deficient mice were surprisingly functional and *Nlrc5*-deficient T cells were targeted by NK cells upon infection, we investigated whether elimination of endogenous *Nlrc5*^*−/−*^ T cells was observed upon infection with LCMV clone 13. We followed the abundance of T cells in the blood over 60 days after infection. Interestingly, CD8^+^ T-cell percentages declined in the blood at late phases of the infection in CD4Cre *Nlrc5*^*fl/fl*^ mice, as illustrated also by flow cytometric plot (Fig. 5a). The reduced abundance of CD8^+^ T cells was confirmed in the spleen of these mice (Fig. 5b). Intriguingly, CD4^+^ T-cell percentages and numbers were only moderately changed (Supplementary Fig. 4a,b). We next looked at whether a similar phenomenon was observed in conventional *Nlrc5*^*−/−*^ mice. These mice have however reduced CD8^+^ T-cell percentages already at steady state^1^, most likely due to diminished MHCI on cells involved in CD8^+^ T-cell selection and/or maintenance. We therefore indicated with a dotted line the expected levels of CD8^+^ T cells in the spleen based on blood percentages measured before the infection, showing that the loss of CD8^+^ T cells is minimal (Supplementary Fig. 4c). We thus evaluated the role of NK cells in the CD8^+^ T lymphocyte loss observed in CD4Cre *Nlrc5*^*fl/fl*^ mice by depleting NK cells from day five after infection, a regimen that allowed establishment of a chronic infection^11^. Remarkably, CD8^+^ T cells were rescued (Fig. 5a,b), suggesting that *Nlrc5*-deficient CD8^+^ T cells were reduced by NK-cell-mediated elimination.

Importantly, in CD4Cre *Nlrc5*^*fl/fl*^mice, a similar decrease was observed both in naive and CD44^hi^-activated CD8^+^ T cells, and rescued by NK-cell depletion completely or partially, respectively (Fig. 5c). Reflecting data on CD44^hi^ effector CD8^+^ T cells, a strong reduction and partial rescue of gp33-specific T cells was observed (Fig. 5d). In agreement with the strongly decreased CD8^+^ T cells, viral burdens were remarkably higher in CD4cre *Nlrc5*^*fl/fl*^ mice and although NK depletion partly rescued effector CD8^+^ T cells, this was not sufficient for virus control (Supplementary Fig. 4d). Altogether, these data demonstrate the existence of two key functions for NLRC5 in antiviral T-cell responses, one intrinsic to rare exhausted antigen-specific effector cells and one, crucial for the entire CD8^+^ T-cell population, clearly dependent on NK cells.

To detail the molecular pathways leading to the observed NK-cell-mediated loss of CD8^+^ T cells, we thoroughly analysed the expression of NK-cell receptors and ligands on NK and T cells, respectively, in the chronic phase of the infection. NK cells from CD4Cre *Nlrc5*^*fl/fl*^ mice did not exhibit major differences for a panel of activating and inhibitory receptors (Supplementary Fig. 4e–g), suggesting that NK cells were minimally influenced by alterations in T cells. We thus analysed the expression of inhibitory and activating ligands on T cells from infected CD4Cre *Nlrc5*^*fl/fl*^, focusing in particular on NK-depleted mice, in which T lymphocytes otherwise targeted by NK cells are present. Whereas stainings for CD23, CD70, VCAM-1, CD155, E-Cadherin, Mult1 and Rae1δ were undetectable on T cells, this analysis revealed that among the NK-cell receptor ligands expressed, profound differences were measured exclusively for MHCI expression (Fig. 5e). Such differences were kept over the course of the infection, as shown for H2-K on cytotoxic T lymphocytes (Supplementary Fig. 4h). Interestingly, CD8^+^ T cells expressed substantially higher levels of the activating ligand CD54 than CD4^+^ T lymphocytes (Fig. 5e), in both control and CD4Cre *Nlrc5*^*fl/fl*^, suggesting that this factor contributes *per se* to their increased susceptibility to NK-cell-mediated elimination, which is in the case of CD4Cre *Nlrc5*^*fl/fl*^ mice potentiated by the strikingly decreased levels of MHCI^35^.

### CD4cre *Nlrc5^fl/fl^* mice reject ‘self' *Nlrc5* ^*−/−*^ T cells

**Figure 6:**
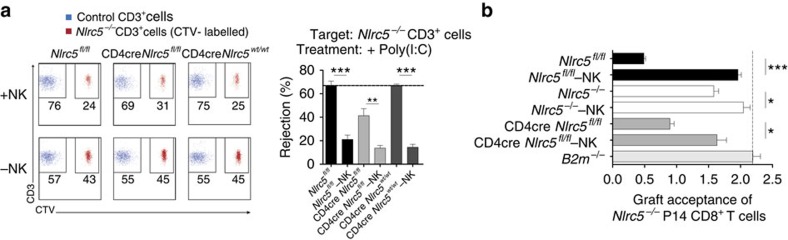
Self-tolerance towards *Nlrc5*^*−/−*^ T cells is broken by NK cells during inflammatory reactions. (**a**) *In vivo* elimination of *Nlrc5*^*−/−*^ CD3^+^ T cells was analysed in the spleen 1 day after transfer into NK cell-depleted or not *Nlrc5*^*fl/fl*^, CD4cre *Nlrc5*^*fl/fl*^ and CD4cre *Nlrc5*^*wt/wt*^ mice, which had been pretreated with Poly(I:C) 1 day before cell transfer. Wild-type splenocytes were co-injected as control. Left panel shows a representative dot plot of control (blue) and *Nlrc5*^*−/−*^ (red) T cell percentages among transferred cells and right panel depicts percentage of rejection for each of the aforementioned mouse strains. Data represent mean±s.e.m. of 3–5 mice per group and results are representative of at least two independent experiments. (**b**) *In vitro* expanded *Nlrc5*^+/−^ and *Nlrc5*^*−/−*^ P14 CD8^+^ T cells were co-transferred into *Nlrc5*^*fl/fl*^, *Nlrc5*^*−/−*^, CD4cre *Nlrc5*^*fl/fl*^, NK-depleted or not and *B2m*^*−/−*^ mice. Data show engraftment of *Nlrc5*^*−/−*^ P14 T cells normalized to the co-injected control cells and to the initial mix. The results represent mean±s.e.m. of *n*=3–4 mice per group. Results are representative of at least two independent experiments. +NK, non-depleted; −NK, depleted of NK cells; *P<0.05; **P<0.01; ***P<0.001; Student's *t*-test.

To further dissect the mechanisms underlying the NK-cell-mediated reduction of CD8^+^ T cells observed upon chronic LCMV infection, we decided to test the rejection of ‘self' *Nlrc5*-deficient T cells by CD4Cre *Nlrc5*^*fl/fl*^ mice in a classical rejection experiment. In resting CD4cre *Nlrc5*^*fl/fl*^ mice, negligible elimination was measured (Supplementary Fig. 5a), in agreement with data presented in Fig. 4a. However, significant rejection was observed in Poly(I:C)-primed CD4Cre *Nlrc5*^*fl/fl*^ mice (Fig. 6a), albeit less as compared with control mice. This was substantially mediated by NK cells, as shown by NK-cell depletion, whose efficiency is shown in Supplementary Fig. 5b. In contrast, negligible NK-cell-mediated rejection of splenic *Nlrc5*^*−/−*^ T cells was measured in Poly(I:C)-primed *Nlrc5*-deficient mice, although in some experiments rejection in the range of 10% was observed (Supplementary Fig. 5c). This indicates that self-tolerance was largely established in *Nlrc5*-deficient mice, consistent with results in chronic LCMV infection. Conversely, NK cells from CD4Cre *Nlrc5*^*fl/fl*^ mice became auto-aggressive under inflammatory conditions.

To corroborate that the activity of NK cells was directly affecting also effector CD8^+^ T cells, we assessed the rejection of *in vitro* activated *Nlrc5*^*−/−*^ and control P14 T cells in Poly(I:C)-primed CD4Cre *Nlrc5*^*fl/fl*^ as well as *Nlrc5*^*−/−*^ mice. Whereas *Nlrc5*^*−/−*^ P14 T cells were considerably rejected in control recipients, efficient engraftment was observed in *Nlrc5*^*−/−*^ mice, supporting the idea that an almost complete tolerance towards self was established in these mice (Fig. 6b). Corroborating our previous data, CD4Cre *Nlrc5*^*fl/fl*^ mice robustly eliminated *Nlrc5*^*−/−*^ effector T cells, largely hindering their engraftment (Fig. 6b). These results thus show that *Nlrc5* deficiency on T cells, reducing but not abolishing MHCI expression, allows NK cells to maintain a high state of responsiveness and recognize endogenous low levels of MHCI as ‘missing-self' in an inflammatory milieu.

## Discussion

We show here the importance of NLRC5 in protecting T cells from NK-cell cytotoxicity and—reciprocally—in shaping NK-cell tolerance and phenotype. In fact, we could show that Ly49I expression is modulated through sensing environmental and NK-cell-intrinsic MHCI levels. This extends previous work showing that high environmental MHCI levels negatively affect NK-cell receptor expression^30^. Mechanistically, these results raise the possibility that MHCI molecules stabilize Ly49I through *cis*-interactions, thereby counteracting their downmodulation in the presence of *trans*-interactions with MHCI^26^. However, the use of NKcre *Nlrc5*^*fl/fl*^ mice showed that NLRC5 expression in NK cells is not essential to maintain responsiveness towards ‘missing-self' targets, suggesting that the observed phenotypical differences are functionally not relevant.

Despite the extremely low MHCI levels exhibited by specific cell subsets, NK cells from *Nlrc5*-deficient mice surprisingly mediated largely effective missing-self responses. This underlines that our model is distinct from previously described mosaic mice, in which MHCI is totally absent on part of the cells^28^,^36^,^37^. Here we found that 10–30% residual MHCI in selected cell subsets is sufficient to maintain NK-cell responsiveness to a large extent, inferring that MHCI levels in wild-type mice are in excess^38^. Along this line, *Nlrc5*-deficient T cells are not eliminated when transferred into resting wild-type hosts, indicating that NK cells tolerate large fluctuations in MHCI levels.

In agreement with studies demonstrating that inflammation increases the state of NK-cell responsiveness^15^,^39^,^40^,^41^, we show that *Nlrc5*^*−/−*^ splenocytes, T cells in particular, were rejected in wild-type hosts upon inflammation or infection. Therefore, NK cells are significantly less tolerant towards partial reductions in MHCI levels under inflammatory conditions, explaining the apparent excess in MHCI expression at steady state and underlining the essential function of NLRC5 in T lymphocytes.

To address a physiologically more relevant question, we wondered in how far elimination of *Nlrc5*^*−/−*^ T cells occurred in an endogenous system. Whereas T-cell-specific *Nlrc5*-deficient mice were unable to establish complete self-tolerance, full knockout animals induced it rather efficiently, in agreement with the notion that the size of MHCI-negative population correlates with tolerance induction^26^. Future research aimed at dissecting the contribution of NLRC5 in additional cell subsets will help detail quantitative and qualitative aspects of this process. Along this line, previous findings demonstrated that lack of MHCI on T cells was sufficient to establish complete NK-cell tolerance towards MHCI-deficient cells^26^. Conversely, our data show that *Nlrc5*^*−/−*^ and CD4Cre *Nlrc5*^*fl/fl*^ mice simultaneously host educated NK cells and potential targets, a prerequisite for breaking self-tolerance. To our surprise, NK cells raised in the presence of *Nlrc5*^*−/−*^ T cells, rejected endogenous *Nlrc5*^*−/−*^ T cells under inflammatory conditions, implying that a clear-cut distinction should be made between low and absent MHCI levels with regard to the establishment of NK-cell tolerance.

Our results also uncovered the existence of two NLRC5-dependent pathways essential for CD8^+^ T-cell maintenance during the chronic phase of LCMV infection in CD4Cre *Nlrc5*^*fl/fl*^ mice. First, NLRC5 sustains rare, exhausted antigen-specific T cells, thereby controlling viral load, an extremely important observation, which deserves future investigations. Second, *Nlrc5* deficiency drives the NK-cell-dependent loss of the entire CD8^+^ T-cell population. Indeed, NK-cell depletion rescued CD8^+^ T cells globally, albeit effector cells to a smaller extent. Their complete rescue was prevented by premature disappearance, which is the dominating effect caused by *Nlrc5* ablation in effector cells. Thus, differently from the commonly held view that NK lymphocytes mainly attack effector T cells^7^,^8^,^9^,^10^,^11^,^12^,^13^,^14^,^15^,^16^,^17^, this phenomenon likewise affects naive and activated subsets, as shown also in classical rejection experiments. Notably, this leads to a generalized CD8^+^ T-cell lymphopenia, which might favour superinfections both by depleting naive T cells and compromising pregressed immunological memory against other pathogens.

Intriguingly, upon LCMV infection, CD4^+^ T-cell numbers were only marginally altered, in spite of markedly reduced MHCI levels^1^,^2^,^6^. Interestingly, expression of the NK-cell-activating ligand CD54 was considerably higher on CD8^+^ T cells, possibly explaining their increased susceptibility. Corroborating the role of MHCI in this setting, the NK-cell-activating ligands tested were only marginally altered by *Nlrc5* deletion, in agreement with our previous data showing in T cells the specificity of NLRC5 for selected MHCI genes genome wide^2^. We therefore speculate that the combined presence of an *Nlrc5*-independent, increased susceptibility to NK-cell-mediated cell death^7^,^8^,^9^,^10^,^11^,^12^,^13^,^14^,^15^,^16^,^17^,^18^ together with the *Nlrc5*-dependent reduced MHCI levels leads to the preferential elimination of CD8^+^ T cells.

Rapid rejection of *in vitro* expanded cytotoxic T cells by CD4Cre *Nlrc5*^*fl/fl*^ mice corroborated the prospect that also activated CD8^+^ T lymphocytes are targets of NK-cell-mediated elimination and can be rescued by NK-cell depletion. Autologous transfer of effector T cells is increasingly used in cancer immunotherapy and often combined with administration of interleukin-2 or other agents inducing inflammation^42^,^43^. Our data suggest that incomplete NK-cell tolerance might affect engraftment efficiency in such settings. Conversely, they also indicate that NK cells can be better exploited in controlling MHCI low tumours and graft-versus leukaemia settings if the right inflammatory milieu is provided, in agreement with previous findings on MHCI-negative tumours^44^. Our work therefore encourages further research determining the impact of these findings on antiviral responses or antitumoural immunotherapies.

MHCI expression strongly varies among different tissues and independent mechanisms regulating it are emerging^45^,^46^. In humans, the range of functional NK-cell receptor–MHCI interactions differs enormously due to the polymorphic nature of these genes^18^,^47^ and the variable levels of MHCI and NLRC5 expression introduce an additional degree of complexity. This intraindividual variability raises the question on how such differing MHCI levels within an individual are considered ‘normal' by NK cells. Elimination of endogenous cells by NK cells has been attributed to the expression of activating receptors on target cells. Yet, our data show that relatively low MHCI expression on endogenous cells concurs to their rejection. *Nlrc5* deficiency thus provides new insights into the fine-tuning of NK-cell tolerance, which are likely to be relevant in modulating immune responses in naturally arising or therapeutically induced inflammatory conditions in humans.

## Methods

### Mice

Control mice (*Nlrc5*^*fl/fl*^) and *Nlrc5*^*−/−*^ (ref. 1), CD45.1^+^ congenic, and *B2m*^*−/−*^ (purchased from Jackson Laboratories) on a C57BL/6 (H2^b^) background were bred in the animal facility of the University of Lausanne. P14αβ mice^48^ were provided by D. Zehn and crossed onto *Nlrc5*^*−/−*^(C57BL/6 background). T- and NK-cell-specific knockout mice for *Nlrc5* were generated by crossing *Nlrc5*^*fl/fl*^ to the *Cd4*cre (purchased from Jackson Laboratories) or the *Ncr1*cre deleter strain^33^, respectively. *Nlrc5*^*−/−*^on BALB/c (H2^d^, purchased from Harlan) background were generated by backcrossing 10 times onto BALB/c in the animal facility of the University of Lausanne. Sex- and age-matched 6–12-week-old mice were used. Mice were treated in accordance with the Swiss Federal Veterinary Office guidelines.

### Cells and tissue preparation

Splenocytes from transgenic P14 mice were grown in RPMI 1640 (Life Technologies) supplemented with 10% fetal calf serum (FCS, from PAA), 100 U ml^−1^ penicillin, 100 μg ml^−1^ streptomycin, 1 mM sodium pyruvate and 50 μM β-mercaptoethanol (all from Life Technologies), and were incubated at 37 °C in 5% CO_2_ with 0.5 μM gp33 peptide (EMC). On day 4, cells were split and human interleukin-2 supplemented at 10 ng ml^−1^. RMA and RMA-S cell lines were maintained in RPMI 1640 supplemented with 10% FCS, 100 U ml^−1^ penicillin, 100 μg ml^−1^ streptomycin and 50 μM β-mercaptoethanol at 37 °C with 5% CO_2_. For the preparation of tissues for qRT–PCR analysis, mice were perfused with Heparin in PBS, organs isolated and put in RNAlater Solution (Ambion), then processed in TriFast reagent (PEQLAB Biotechnologie GmbH) using the TissueLyser according to the manufacturer's instructions (Qiagen).

### Human T- and NK-cell isolation

Peripheral blood mononuclear cells were isolated by density centrifugation over a Ficoll-Hypaque gradient (LymphoPrep) from peripheral blood of healthy human donors. T cells were enriched using a mix of α-CD4 and α-CD8 magnetic beads (Miltenyi Biotech), NK cells were isolated using the NK-cell-negative selection kit from StemCell Technologies (Grenoble, France), according to the manufacturer's recommendations. Untouched NK cells were labelled with a cocktail of anti-CD3 (UCHT1, 1:100), CD14 (HCD14, 1:100), CD19 (HIB19, 1:100), CD56 (HCD56, 1:100) and CD16 (3G8, 1:200) antibodies (all from BioLegend). Live, CD3^−^CD14^−^ CD19^−^, CD56^dim^CD16^pos^ and CD56^bright^CD16^neg^ NK-cell subsets were isolated using fluorescence-activated cell sorting (FACS Aria, BD Biosciences). Purity of isolated cells was always >95%, as assessed by flow cytometry. Buffy coats were purchased from the Blood Transfusion Center, Lausanne, Switzerland, and all subjects gave their written consent.

### Flow cytometry

For flow cytometry analysis, cells were preincubated with α-CD16/32 (2.4G2, 1:100) to block Fc receptors and then surface stained using antibodies against CD3e (145-2C11, 1:100), CD4 (L3T4, 1:300), CD8a (Ly-2, 1:600), CD11a (M17/4, 1:150), CD11b (M1/70, 1:250), CD11c (N418, 1:150), CD16/32 (93, 1:400), CD18 (M18/2, 1:100), CD19 (1D3, 1:200), CD23 (B3B4, 1:100), CD27 (LG.7F9, 1:200), CD29 (eBioHMb1-1, 1:100), CD44 (IM7, 1:200), CD45.1 (A20, 1:100), CD45.2 (104, 1:100), CD49d (R1-2, 1:100), CD62L (MEL-14, 1:300), CD48 (HM48-1, 1:150), CD54/ICAM-1 (YN1/1.7.4, 1:100), CD70 (FR70, 1:100), CD94 (18D3, 1:150), CD102/ICAM-2 (3CA, 1:100), CD106/VCAM-1 (429, 1:100), CD122 (TM-b1, 1:100), CD155 (TX56, 1:150), CD226/DNAM (10E5, 1:150), CD244/2B4 (eBio244F4, 1:150), CD305 (113, 1:150), B220 (RA3-6B2, 1:200), DX5/CD49b (DX5, 1:100), E-Cadherin (DECMA-1, 1:150), H2-D^b^ (28-14-8, 1:250), H2-K^b^ (AF6-88.5.5.3, 1:250), H2-K^d^/D^d^ (34-1-2S, 1:150), Klrg1 (2F1, 1:200), Ly49A (A1, 1:100), Ly49D (4E5, 1:100), Ly49G2 (4D11, 1:300), Ly49I (YLI-90, 1:100), Mult1 (5D10, 1:75), NKG2D (CX5, 1:100), NKG2A/C/E (20d5, 1:100), NK1.1 (PK-136, 1:100), NKp46 (29A1.4, 1:100), Qa2 (69H1-9-9, 1:200), Rae1δ (RD-41, 1:75) (all from eBioscience), H2-L^d^ (28-14-8, 1:200), Ly49H (3D10, 1:100) and Ly49C/I (5E6, 1:100; from BioLegend). The H-2D^b^–gp33 tetramer (1:100) was from TCMetrix. Streptavidin conjugated to different fluorophores were from eBioscience. Stainings were performed with appropriate combinations of fluorophores. Data were acquired with a Becton Dickinson flow cytometer and analysed using FlowJo software (Tree Star).

### Quantitative RT–PCR analysis

Total RNA was extracted using the TriFast reagent according to the manufacturer's instructions (PEQLAB Biotechnologie GmbH). Annealing with random primers (Life technologies) was performed at 70 °C for 5 min, followed by retrotranscription to complementary (cDNA) with M-MLV RT, RNase H(–) point mutant (Promega) and nucleotides (Roche Diagnostics) by incubating at 40 °C for 10 min, 45 °C for 50 min and 70 °C for 15 min. cDNA was purified with the Wizard SV gel and PCR clean-up system following the manufacturer's instructions (Promega).

cDNA was quantified using the LightCycler 480 SYBR Green I Master (Roche Diagnostics) on a LightCycler 480 machine (Roche Diagnostics). Standard cycling was used (45 cycles of 95, 60 and 72 °C of 10 s each). Expression was determined relative to the housekeeping genes as indicated. Data were analysed, and transcript abundance (gene/housekeeping gene) and s.d. were calculated using the LightCycler 480 software.


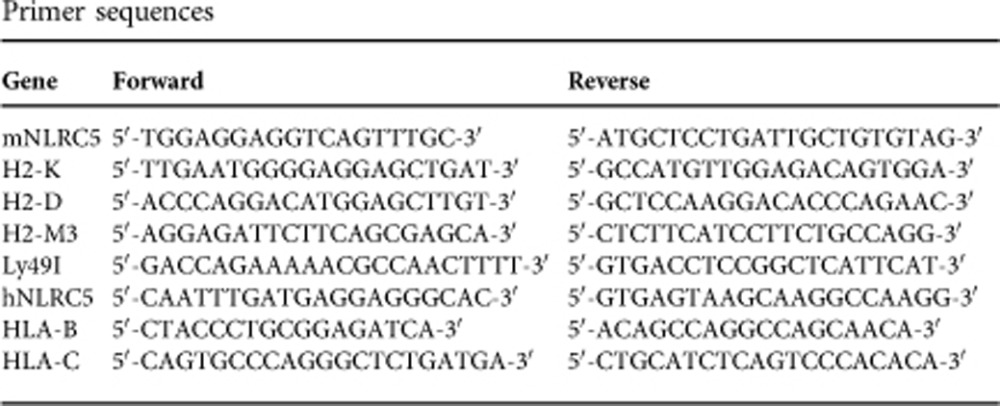


### Acidic treatment to assess masking of NK-cell receptors

Cells were washed twice in PBS and resuspended for 1 min at room temperature in 1 ml citrate buffer (0.133 M citric acid and 0.066 M Na_2_HPO_4_, pH 3.3). Treatment was stopped by adding an excess of medium. After washing, cells were stained for flow cytometry as detailed above.

### *In vivo* NK-cell-mediated rejection

Recipient mice were pretreated or not 1 day before cell transfer with 100 μg Poly(I:C) (InvivoGen) by intraperitoneal injection. Then, 5–10 × 10^6^ splenocytes from *B2m*-deficient, *Nlrc5*-deficient and CD45.1^+^ wild-type mice were injected intravenously into recipient mice. To distinguish transferred target cell populations, labelling of cells with carboxyfluorescein succinimidyl ester (CFSE, Sigma) and CellTrace Violet (CTV, Life Technologies) was performed in PBS 1% FCS at 37 °C at 2.5 μM for 8 min and 2 μM for 20 min, respectively. Loss of target cells in the spleen was analysed 1 day (with Poly(I:C) pretreatment) or 2 days (without pretreatment) after cell transfer, and is shown as percentage of rejection normalized to the co-injected wild-type cells and to the initial mix. In some mice, NK cells were depleted by intraperitoneal injection of 200 μg of α-NK1.1 antibody (PK-136, purchased from BioXcell) 1–2 days before cell transfer.

### Tumour clearance *in vivo*

Mice were co-injected intraperitoneally with 10^6^ RMA (MHCI^+^) and 10^6^ RMA-S (MHCI^-^) labelled with CFSE and CTV as described above. *In vivo* tumour clearance was assessed at day 2 after transfer by flow cytometry in peritoneal lavage. In some mice, NK cells were depleted as described above.

### Adoptive transfer of P14 CD8^+^ T cells and LCMV infection

Transgenic P14 CD8^+^ T cells were isolated with α-CD8a magnetic beads (Miltenyi Biotech). Control (2 × 10^3^; *Nlrc5*^*wt/−*^) and 2 × 10^3^ *Nlrc5*^*−/−*^ P14 CD8^+^ T cells were intravenously co-transferred into naive *Nlrc5*^*fl/fl*^ mice. In some mice, NK cells were depleted as described above. The LCMV clone 13 strain was propagated according to an established protocol^49^. Frozen stocks were diluted in PBS and 2 × 10^6^ plaque-forming units were injected intravenously into mice. For adoptive transfer experiments with P14 cells, mice were infected 1 day after cell transfer. For persistent infection of *Nlrc5*^*fl/fl*^ and CD4Cre *Nlrc5*^*fl/fl*^ mice, NK1.1 depletion by intraperitoneal injection of 200 μg of α-NK1.1 was started on day 5 after infection and repeated every 7–9 days. Splenocyte suspensions from LCMV-infected mice were ‘shock frozen' to release the virus. Diluted samples were used for the infection of Vero cells, and viral titres were determined by an LCMV plaque-forming assay^49^.

### Mixed bone marrow chimeras

Recipient mice were depleted of NK cells as described above. BM from sex-matched donor mice was obtained by flushing femurs and tibias and was mixed in a 1:1 ratio and a total of 8 × 10^6^ BM cells were injected intravenously into recipient mice, which had been lethally irradiated with 900 rad. Mice were analysed not earlier than 6 weeks after reconstitution.

### NK-cell transfer

Splenic NK cells were enriched using negative selection by MACS (CD19^−^CD3^−^; Miltenyi Biotech) or by the NK-cell isolation kit (Stemcell Technologies), labelled with CFSE and CTV as described above, and ∼5 × 10^5^ cells of each genotype injected intravenously into recipient mice. After 24 h, transferred NK cells were analysed for the expression of the NK-cell receptor repertoire.

### Statistical analysis

Statistical analyses were performed using Prism software (GraphPad version 5.0). The Student's *t*-test (unpaired, two tailed) was used to compare the significance of differences between experimental groups. Correlations were evaluated using Pearson correlation analysis. Differences were considered significant when *P*<0.05 (*), very significant when *P*<0.01 (**) and highly significant when *P*<0.001 (***).

## Additional information

**How to cite this article:** Ludigs, K. *et al*. NLRC5 shields T lymphocytes from NK-cell-mediated elimination under inflammatory conditions. 7:10554 doi: 10.1038/ncomms10554 (2016).

## Supplementary Material

Supplementary Information Supplementary Figures 1-5
