# Supplementary material for: NLRC5 shields T lymphocytes from NK-cell-mediated elimination under inflammatory conditions
Source: Nat Commun. 2016 Feb 10;7:10554. doi: 10.1038/ncomms10554 (PMC4749981; doi:10.1038/ncomms10554)
Supplement: Supplementary Information — Supplementary Figures 1-5 [file ncomms10554-s1.pdf]

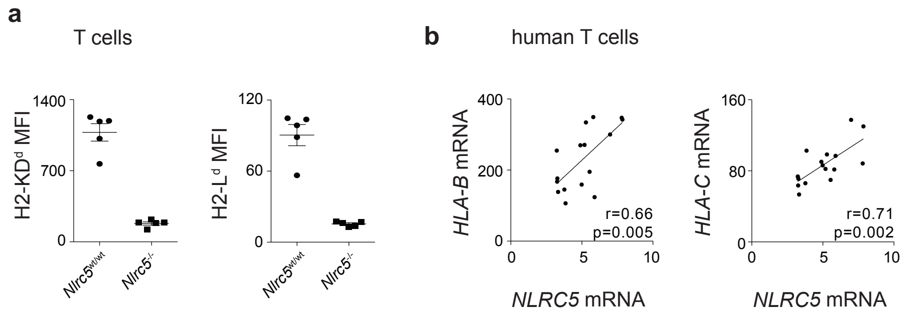

Supplementary Figure 1. *NLRC5* expression and regulation of H2-L and *HLAs*.
(a) H2-KD^d and H2-L^d expression was analyzed by flow cytometry on splenic T cells ($CD3^+$) from *Nlrc5*$^{wt/wt}$ and *Nlrc5*$^{-/-}$ mice on BALB/c background. Mean ± SEM of MFIs for H2-KD^d and H2-L^d of n = 5 mice per group are illustrated.
(b) Correlation between *HLA-B* or *HLA-C* mRNA with *NLRC5* mRNA expression (relative to the housekeeping genes *PGK*, *PPIA*, and *POLR2A*) are shown in human healthy donor-derived T cells (n=16).

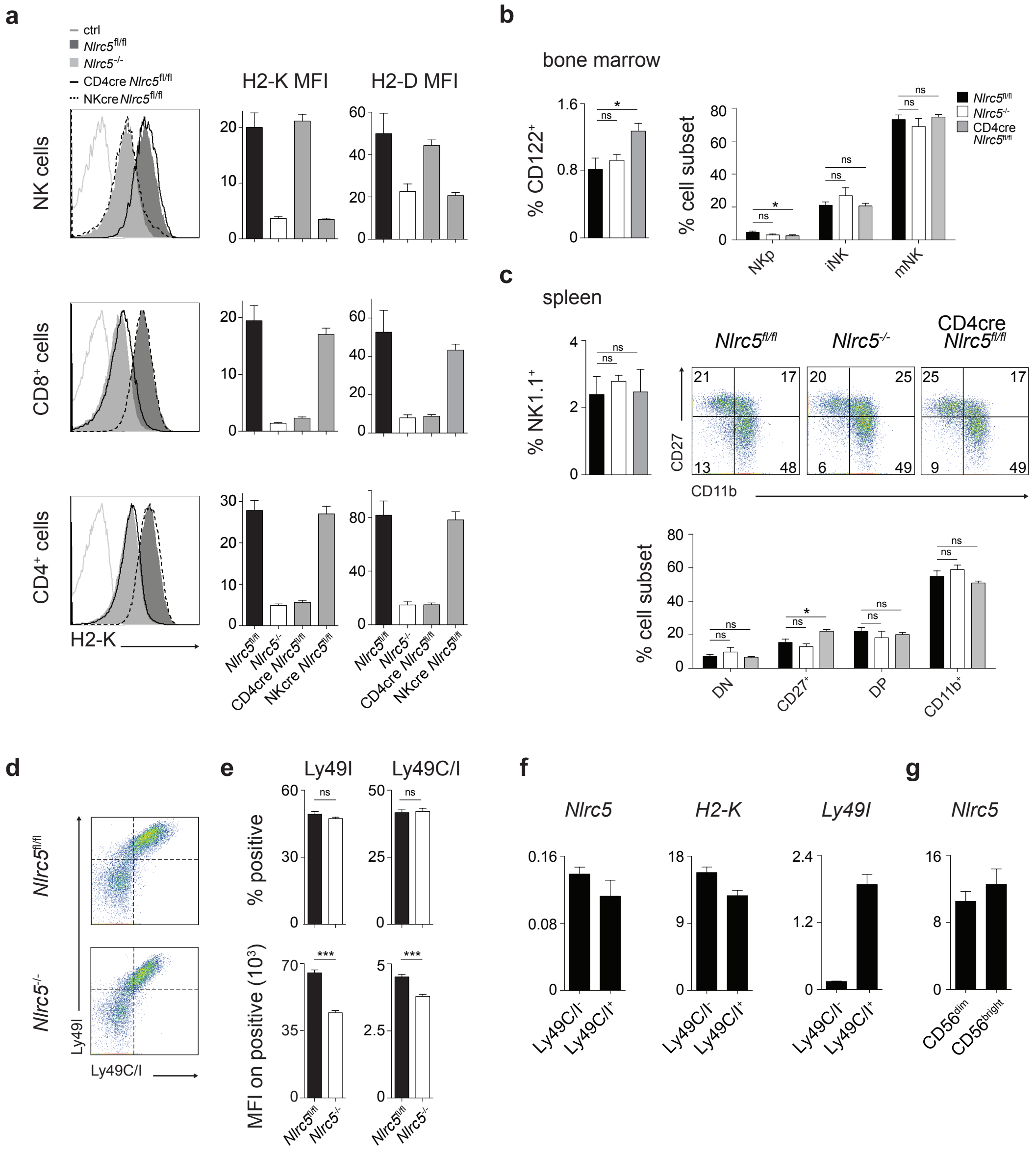

Supplementary Figure 2. CD4cre and NKcre *Nlrc5*$^{fl/fl}$ mice and NK cell characterization.

(a) The histogram overlay shows a representative mouse from *Nlrc5*$^{fl/fl}$, *Nlrc5*$^{-/-}$, CD4cre *Nlrc5*$^{fl/fl}$, and NKcre *Nlrc5*$^{fl/fl}$ strains for H2-K expression. H2-K and H2-D expression were assessed on splenic NK cells, CD8^{+}, and CD4^{+} T cells. Mean ± SEM of MFIs of n = 4-6 mice per group are illustrated. (b) Left panel: percentages of CD122^{+} cells are shown in the BM for *Nlrc5*$^{fl/fl}$, *Nlrc5*$^{-/-}$, and CD4cre *Nlrc5*$^{fl/fl}$ mice. Right panel: the development of NK cells is depicted as percentages of precursor (NKp, CD3^{-}CD122^{+}NK1.1^{-}DX5^{-}), immature (iNK, CD3^{-}CD122^{+}NK1.1^{+}DX5^{-}), and mature NK cells (mNK, CD3^{-}CD122^{+}NK1.1^{+}DX5^{+}) in the BM as analyzed in the afore-mentioned genotypes. (c) Left panel: percentages of NK cells (NK1.1^{+}CD3^{-}CD19^{-}) are depicted in the spleen for *Nlrc5*$^{fl/fl}$, *Nlrc5*$^{-/-}$, and CD4cre *Nlrc5*$^{fl/fl}$ mice. Representative cytometric profiles of splenic NK cells stained with CD27 and CD11b (upper panel) and percentages of double-negative (DN, CD27^{-}CD11b^{-}), CD27 single-positive (CD11b^{-}CD27^{+}), double-positive (DP, CD27^{+}CD11b^{+}), and CD11b single-positive (CD27^{-}CD11b^{+}) populations (lower panel) are shown. Results represent mean ± SEM (n = 6-8 mice per group) and represent the pool of two independent experiments (b, c). (d) Representative cytometric profiles of splenic NK cells stained for Ly49I and Ly49C/I in *Nlrc5*$^{fl/fl}$ and *Nlrc5*$^{-/-}$ mice. (e) Graphs depict percentages of Ly49I^{+} and Ly49C/I^{+} NK cells and MFI of Ly49I and Ly49C/I on the positive population for *Nlrc5*$^{fl/fl}$ and *Nlrc5*$^{-/-}$ mice. Results represent mean ± SEM (n = 4 mice per group) and are representative of three independent experiments (e). (f) *Nlrc5*, *H2-K*, and *Ly49I* mRNA expression was quantified relative to *Hprt* mRNA in FACS-sorted, CD3^{-}CD19^{-}NK1.1^{+}, Ly49C/I^{-} or Ly49C/I^{+} NK cells. Results depict mean ± SD (n = 3 replicates). (g) *Nlrc5* mRNA expression in FACS-sorted CD56^{dim} and CD56^{bright} NK cells from healthy human donors. Results represent mean ± SEM (n = 3 donors).

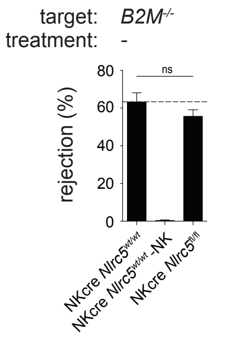

Supplementary Figure 3. ***B2m**$^{-/-}$* **splenocytes are rejected by NKcre** ***Nlrc5**$^{fl/fl}$* **mice.**
Rejection of *B2m*$^{-/-}$ as compared to wild type splenocytes was analyzed in the spleen of NK cell-depleted or not NKcre *Nlrc5*$^{wt/wt}$ and NKcre *Nlrc5*$^{fl/fl}$ mice two days after transfer. Wild type splenocytes were co-injected as control. Data is shown as percentage of rejection and represent mean ± SEM of n = 4 mice per group and results are representative of at least two independent experiments.

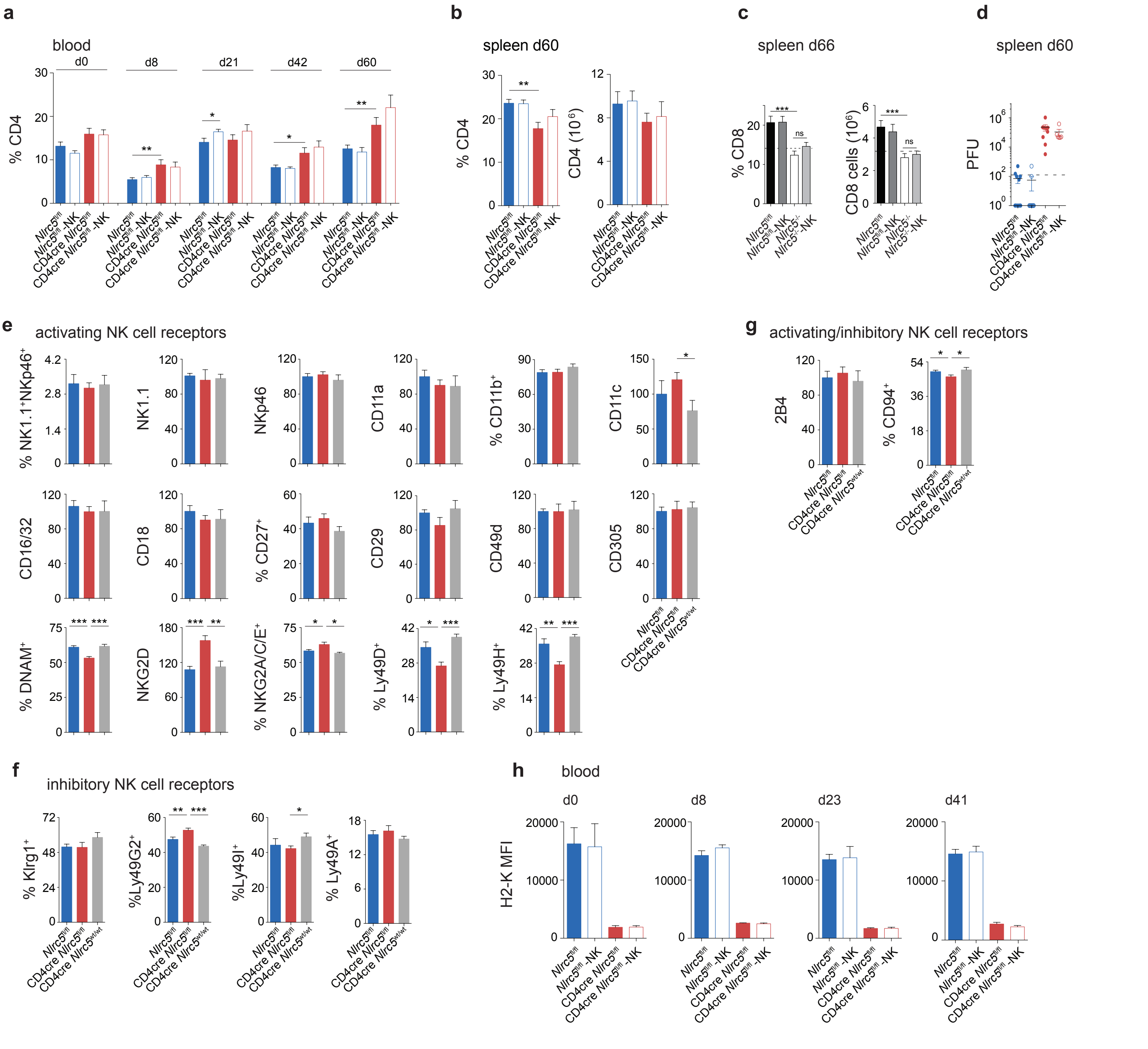

Supplementary Figure 4. Further characterization of T and NK cells of CD4cre *Nlrc5*$^{fl/fl}$ mice in infected conditions.
(a) Percentages of CD4$^+$ T cells (gated on lymphocytes) in the blood over the course of LCMV clone 13 infection are illustrated for NK cell -depleted or not *Nlrc5*$^{fl/fl}$ and CD4cre *Nlrc5*$^{fl/fl}$ mice. (b) Percentages and absolute numbers of splenic CD4$^+$ T cells are depicted at day 60 p.i.. Results show a pool of two experiments representing mean ± SEM (n = 7-13 mice per group) and are representative of three independent experiments (a, b). Statistical differences are depicted between CD4cre *Nlrc5*$^{fl/fl}$ and *Nlrc5*$^{fl/fl}$ or NK-depleted CD4cre *Nlrc5*$^{fl/fl}$, respectively, when significant (a, b). (c) Percentages and absolute numbers of splenic CD8$^+$ T cells in *Nlrc5*$^{fl/fl}$ and *Nlrc5*$^{-/-}$ mice, depleted or not of NK cells, are depicted for day 66 p.i.. Results show a pool of two experiments representing mean ± SEM (n = 9-11 mice per group) and are representative of four independent experiments. Dotted line indicates the expected percentage of CD8$^+$ T cells in *Nlrc5*$^{-/-}$ mice. Statistical differences are depicted between *Nlrc5*$^{-/-}$ and *Nlrc5*$^{fl/fl}$ or NK-depleted *Nlrc5*$^{-/-}$, respectively (c). (d) LCMV clone 13 viral titers in the spleens at day 60 p.i. of NK cell-depleted or not *Nlrc5*$^{fl/fl}$ and CD4cre *Nlrc5*$^{fl/fl}$ mice. PFU, plaque-forming units; dotted line, limit of detection. Results show a pool of two experiments showing mean ± SEM (n = 6-13 mice per group). (e-g) Percentages of NK cells and expression analysis of the indicated activating (e), inhibitory (f), and dual function receptors (g) on NK cells (NK1.1$^+$CD3$^-$CD19$^-$, for NK1.1 expression on NKp46$^+$CD3$^-$CD19$^-$) as percentage of positive population (for biphasic expression) or as MFI (average MFI of control mice was set at 100%) in the spleen of *Nlrc5*$^{fl/fl}$, CD4cre *Nlrc5*$^{fl/fl}$, and CD4cre *Nlrc5*$^{wt/wt}$ mice infected with LCMV for 48 days. Results represent mean ± SEM (n = 6-10), are the pool of two independent experiments, and only significant differences are depicted (e-g). (h) H2-K expression as MFI was analyzed on CD8$^+$ cells during the course of an LCMV infection in the blood of *Nlrc5*$^{fl/fl}$ and CD4cre *Nlrc5*$^{fl/fl}$, depleted or not of NK cells. Results represent mean ± SEM (n = 3-6) and are representative of two independent experiments. -NK, depleted of NK cells.

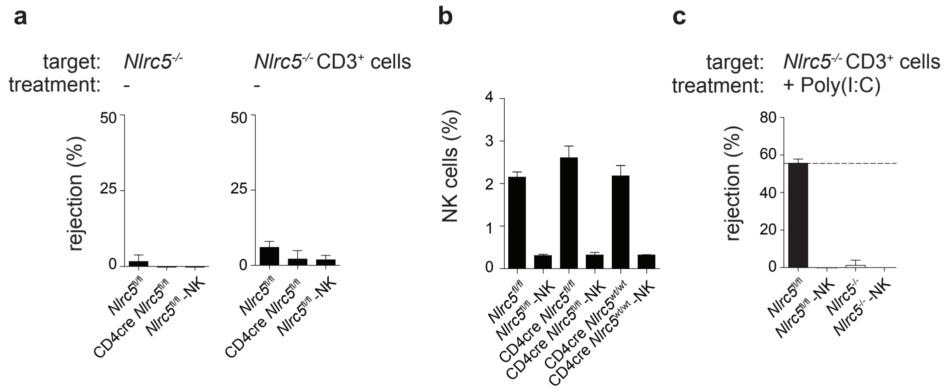

Supplementary Figure 5. Rejection of *Nlrc5*-deficient T cells in CD4cre *Nlrc5$^{fl/fl}$* and *Nlrc5$^{-/-}$* mice.
(a) Rejection of *Nlrc5$^{-/-}$* splenocytes (left panel) and *Nlrc5$^{-/-}$* CD3^{+} T cells (right panel) was analyzed in the spleen two days after transfer into *Nlrc5$^{fl/fl}$*, *CD4cre Nlrc5$^{fl/fl}$*, and NK cell-depleted *Nlrc5$^{fl/fl}$* mice. (b) NK cell percentages were analyzed in the spleen of NK cell-depleted or not *Nlrc5$^{fl/fl}$*, *CD4cre Nlrc5$^{fl/fl}$*, and *CD4cre Nlrc5$^{wt/wt}$* mice, in which rejection of *Nlrc5$^{-/-}$* CD3^{+} T cells was analyzed (Fig. 6a). (c) *In vivo* killing of *Nlrc5$^{-/-}$* CD3^{+} T cells was analyzed in the spleen one day after transfer into NK cell-depleted or not *Nlrc5$^{fl/fl}$* and *Nlrc5$^{-/-}$*, pretreated with Poly(I:C) one day before cell transfer. Wild type splenocytes were co-injected as control (a-c). Data is shown as percentage of rejection and represent mean ± SEM of n = 4 mice per group (a) and results are representative of at least two independent experiments (a) and n = 12-15 mice per group and is a pool of two experiments (c).
